# Association between distorted body image and changes in weight status among normal weight preadolescents in Japan: a population-based cohort study

**DOI:** 10.1186/s13690-016-0151-y

**Published:** 2016-09-20

**Authors:** Takako Shirasawa, Hirotaka Ochiai, Hinako Nanri, Rimei Nishimura, Keiichiro Ikeda, Hiromi Hoshino, Akatsuki Kokaze

**Affiliations:** 1 Department of Public Health, Showa University School of Medicine, 1-5-8 Hatanodai, Shinagawa-ku, Tokyo, 142-8555 Japan; 2 Division of Diabetes, Metabolism and Endocrinology, Department of Internal Medicine, Jikei University School of Medicine, Tokyo, Japan

**Keywords:** Distorted body image, Overweight, Underweight, Preadolescents, Japanese

## Abstract

**Background:**

Distorted body image may be important risk factors for being underweight and overweight. The objective of this study was to investigate the association between having a distorted body image and being overweight or underweight among normal weight preadolescents in a population-based cohort study in Japan for each sex.

**Methods:**

The study participants were 1431 normal weight fourth-grade students (age range: 9–10 years) in Ina town, Japan from 2002 to 2007. The height and weight of each student were measured while they were in the fourth grade (at baseline) and seventh grade (3 years later). Childhood underweight and overweight were defined using the body mass index cut-off points proposed by the International Obesity Task Force. Information regarding the self-perceived weight status of each student at baseline was collected using a self-administered questionnaire. Children who were normal weight but perceived themselves as heavy or thin were regarded as having a distorted body images. A logistic regression model was used to calculate the odds ratios (ORs) and 95 % confidence interval (95 % CI) for being overweight or underweight 3 years later among those having a distorted body image at baseline.

**Results:**

Both boys and girls who perceived themselves to be heavy at baseline were at a statistically significantly greater risk of being overweight 3 years later as compared to boys and girls, respectively, who identified as being at a normal weight at baseline (boys: adjusted OR: 4.66, 95 % CI: 1.01–21.48; girls: 3.88, 1.56–9.65). Both boys and girls who perceived oneself to be thin at baseline were at a statistically significantly greater risk of bring underweight 3 years later as compared to boys and girls, respectively, who identified as being at a normal weight at baseline (boys: 5.51, 2.20–13.80; girls: 2.93, 1.40–6.11).

**Conclusion:**

The results of the present study suggest that having a distorted body image in preadolescence is associated with being overweight or underweight in adolescence, among boys and girls, separately. Therefore, education regarding self-perceived weight could be important to help prevent underweight and overweight/obesity among preadolescent boys and girls in Japan.

## Background

Overweight and obesity have been dramatically increasing among children and adolescents in economically developed countries and urban populations [1]. In Japan, the prevalence of obese boys and girls increased from 6.1 and 7.1 %, respectively, in the time-period 1976 to 1980, to 11.1 and 10.2 % in 1996 to 2000 [2]. Moreover, recent studies have reported that the prevalence of thinness among Japanese adolescents boys and girls have progressively increased from 2.8–4.7 and 2.0–5.7 % in 2003–2004, respectively, to 5.1–7.6 and 3.5–7.8 % in 2011–2012 [3, 4]. Thinness, overweight and obesity among children and adolescents can lead to adverse health effects [5, 6], and should therefore be considered serious public health problems.

Several cross-sectional studies have reported an association between distorted body image and weight status among adolescents [7–10]. For instance, Saleem et al. showed that underweight females tend to overestimate their weight, whereas overweight males tend to underestimate their weight in Pakistan [7]. Perkins et al. reported that among both male and female students in UK, overestimating and underestimating peer weight norms were associated with a greater risk for being overweight and underweight, respectively [8]. Moreover, an association between self-perceived overweight in normal weight adolescents and overweight or obesity later in life has been reported in a few prospective studies [11, 12]. However, to the best of our knowledge, no longitudinal studies have examined the association between having a distorted body image in preadolescence boys and girls and the development of overweight or underweight. In addition, the gender difference of body image among adolescents has been reported; adolescent boys tended to underestimate their body weight, whereas adolescent girls were more likely to overestimate their body image [10, 13–15]. Therefore, to help prevent the development of overweight or underweight, the effect of having a distorted body image among preadolescents on developing overweight or underweight later in life should be prospectively investigated for each sex.

We hypothesized that self-perceived weight status in preadolescence boys and girls would be associated with being overweight or underweight in adolescence for each sex, after controlling for confounding factors that have been reported in previous studies [10, 16]. Accordingly, in this study, we prospectively investigated whether having a distorted body image was associated with being overweight/obese or underweight among normal weight preadolescent boys and girls, separately, in Japan.

## Methods

### Participants

In addition to the annual national health checkups performed in accordance with the School Health Law of Japan, the town of Ina in Saitama Prefecture, Japan, provides a unique health program as part of its community health services. This additional program consists of a questionnaire survey and physical examinations for fourth- and seventh-grade students. The present study was conducted as part of this program.

The participants in the present study were all fourth-grade students (baseline) (age range: 9–10 years) in four Ina town’s elementary schools from 2002 to 2007 (*N* = 2285). Of 2285 fourth-grade students, 2268 participated in this study (participation rate: 99.3 %) from 4 schools with an average school size of 113 students every year (minimum of 71 students and maximum of 165 students). Among the 2268 participants, 2052 (1044 boys and 1008 girls) were followed up in the seventh grade (follow-up rate: 90.5 %). Relocation and absence from the health checkups at seventh grade were the major reasons that students were lost to follow-up. There were no statistical differences between those who were followed up and those who were lost to follow-up in gender (*P* = 0.908) and body mass index (BMI) (*P* = 0.547) at baseline. Of the 2052 children followed up, 621 were excluded from analysis due to being overweight/obese or underweight at baseline (*n* = 516) or because of missing data on self-perceived weight status (*n* = 105). Therefore, a total of 1431 normal weight children (723 boys and 708 girls) were analyzed in the present study.

### Anthropometric measurements

Height and weight measurements were taken for each child in the fourth grade (baseline), and follow-up measurements were carried out in the seventh grade (3 years later). The same examination protocol was used at baseline and 3 years later to ensure uniformity. For the measurements, all children were asked to remove their shoes and socks. Next, their height and weight were measured in increments of 0.1 cm and 0.1 kg, respectively, while they were wearing light clothing. BMI was calculated as body weight (kg) divided by the square of the height (m^2^). Childhood underweight, overweight, and obesity were defined using the BMI cut-off points proposed by the International Obesity Tack Force (i.e., age- and sex-specific cut-off points that were linked to BMI values of 18.5, 25, and 30 at age 18, respectively) [17, 18]. The BMI cut-off points were based on averaged data from six different countries, including Asian countries; these cut-off points have been shown to be applicable to Japanese children [19]. The BMI cut-off points have been used in numerous studies in Japan [3, 4, 10, 19, 20]. In the present study, the BMI cut-off points for childhood underweight, overweight, and obesity were 14.35, 19.10, and 22.77 (age 9), and 14.64, 19.84, and 24.00 (age 10), 15.35, 21.22, and 26.02 (age 12) and 15.84, 21.91, and 26.84 (age 13) for boys, and 14.28, 19.07, and 22.81 (age 9), and 14.61, 19.86, and 24.11 (age 10), 15.62, 21.68, and 26.67 (age 12) and 16.26, 22.58, and 27.76 (age 13) for girls, respectively. For the purposes of the present study, obesity and overweight were included in the same category. Children who were neither underweight nor overweight were regarded as normal weight.

### Questionnaire survey

A self-reported questionnaire survey composed of the following items was conducted on each students at baseline: sex; age; snacking after dinner; eating speed; exercise other than physical education class; and self-perceived weight status. These factors were collected and used as covariates in this study, because lifestyle factors have previously been shown to be associated with perceived body image [10, 16]. Self-perceived weight status was assessed using the question “Do you think you are very thin, thin, normal weight, heavy, or very heavy?”. Because the numbers in “very thin” and “very heavy” were small, perceived weight status was categorized into one of the following three groups for analysis: thin (“very thin” and “thin”); normal (“normal weight”); or heavy (“heavy” and “very heavy”). Children who were normal weight and perceived themselves as heavy or thin were regarded as those who had distorted body images. In addition, the parents or guardians of each student were asked to complete a self-administered questionnaire regarding their child’s wake-up time and bedtime. Data for wake-up time and bedtime were then used to calculate sleep duration. The questionnaire survey was conducted before anthropometric measurements.

### Statistical analysis

The unpaired *t*-test or chi-square test was used to compare characteristics between boys and girls. In analysis stratified by sex, a logistic regression model was used to examine the relationship between distorted body image at baseline and overweight or underweight 3 years later. The odds ratios (ORs) and 95 % confidence interval (95 %CI) for being overweight or underweight 3 years later were estimated and subsequently adjusted for potential confounders, which included exercise, eating speed, snacking after dinner, and hours of sleep at baseline. A *P* value of less than 0.05 was considered statistically significant. All data were analyzed using SPSS 20.0 J (IBM, Chicago, IL, USA).

## Results

The baseline characteristics of the study participants are shown in Table 1. No statistically significant differences were found between boys and girls in height or weight, but BMI was higher in boys than in girls (*P* = 0.004). Moreover, a statistically significant difference was observed between boys and girls in self-perceived weight status (*P* < 0.001); a higher proportion of girls perceived themselves as heavy, whereas a higher proportion of boys perceived themselves as thin. Among the participants, 2.2 and 3.5 % of the boys and 3.8 and 5.1 % of the girls who had been normal weight at baseline were overweight and underweight 3 years later, respectively (data not shown).

**Table 1:** Baseline characteristics of study participants by sex (Japan, 2002–2007) (*n*=1431)

Variable	Boys	Girls	*P*-value
	(*n*=723)	(*n*=708)	
Age (years), mean (SD)	9.4 (0.5)	9.4 (0.5)	0.961
Height (cm), mean (SD)	134.8 (5.4)	134.8 (6.2)	0.968
Weight (kg), mean (SD)	30.3 (3.7)	29.9 (4.1)	0.129
BMI (kg/m^2^), mean (SD)	16.6 (1.3)	16.4 (1.3)	0.004
Seif-perceived weight status, *n* (%)			
Thin	207 (28.6)	151 (21.3)	0.001
Normal	480 (66.4)	498 (70.3)	
Heavy	36 (5.0)	59 (8.3)	

The unpaired t-test and chi-square test were used to compare characteristics between sexes

*SD* standard deviation, *BMI* body mass index

The ORs estimating the relationship between self-perceived weight status at baseline and overweight 3 years later, stratified by gender, are presented in Table 2. Both boys and girls who perceived oneself to be heavy at baseline were at a statistically significantly greater risk of being overweight or obese 3 years later as compared to boys and girls, respectively, who identified as being at a normal weight at baseline (boys: adjusted OR: 4.66, 95 % CI: 1.01–21.48; girls: 3.88, 1.56–9.65). In this analysis, there were no students who perceived themselves as thin at baseline and were overweight 3 years later both boys and girls.

**Table 2:** Associations between self-perceived weight status at baseline and overweight 3 years later (Japan; 2002–2007) (*n*=1431)

	Total	Overweight	Crude	Adjusted^a^
	*N*	*n* (%)	OR (95 % CI)	OR (95 % CI)
Self-perceived weight status				
Boys				
Thin	207	0 (0.0)	-	-
Normal	480	12 (2.5)	1.00	1.00
Heavy	36	4 (11.1)	4.79 (1.46–15.70)	4.66 (1.01–21.48)
Girls				
Thin	151	0 (0.0)	-	-
Normal	498	19 (3.8)	1.00	1.00
Heavy	59	8 (13.6)	3.81 (1.59–9.13)	3.88 (1.56–9.65)

*OR* odds ratio, *95 %* CI 95 % confidence interval

^a^Adjusted for age, exercise, eating speed, snacking after dinner, and hours of sleep at baseline

Next, the ORs estimating the relationship between self-perceived weight status at baseline and underweight 3 years later, stratified by gender, are presented in Table 3. Both boys and girls who perceived oneself to be thin at baseline were at a statistically significantly greater risk of being underweight 3 years later as compared to boys and girls, respectively, who identified as being at a normal weight at baseline (boys: adjusted OR: 5.51, 2.20–13.80; girls: 2.93, 1.40–6.11). In this analysis, there were no students who perceived themselves as heavy at baseline and were underweight 3 years later both boys and girls.

**Table 3:** Associations between self-perceived weight status at baseline and underweight 3 years later (Japan; 2002–2007) (*n*=1431)

	Total	Underweight	Crude	Adjusted^a^
	*N*	*n* (%)	OR (95 % CI)	OR (95 % CI)
Self-perceived weight status				
Boys				
Thin	207	17 (8.2)	5.15 (2.18–12.12)	5.51 (2.20–13.80)
Normal	480	8 (1.7)	1.00	1.00
Heavy	36	0 (0.0)	-	-
Girls				
Thin	151	18 (11.9)	3.47 (1.75–6.85)	2.93 (1.40–6.11)
Normal	498	18 (3.6)	1.00	1.00
Heavy	59	0 (0.0)	-	-

*OR* odds ratio, *95 %* CI 95 % confidence interval

^a^Adjusted for age, exercise, eating speed, snacking after dinner, and hours of sleep at baseline

## Discussion

The results of the present study showed that, compared to those students who perceived themselves as normal weight in fourth grade, students who perceived themselves as heavy had a greater risk of being overweight in seventh grade, among boys and girls, separately. This association remained after controlling for age and lifestyle factors such as exercise, eating speed, snacking after dinner and hours of sleep at baseline. Cuypers et al. reported that adolescents (age 13–19 years) who perceived themselves as overweight but were defined as normal weight had greater weight gain into young adulthood than those who did not perceive themselves as overweight in Norway [11]. Duong et al. reported that youths (age 11–17 years) who perceived themselves as overweight at baseline were approximately 2.5 times more likely than those who perceived themselves as normal weight to be overweight or obese 6 years later in USA [12]. These results from the present study are compatible with these findings.

One explanation for the results of our study is that the perception of being overweight among preadolescents might lead to unhealthy behaviors that result in weight gain. Several previous studies have reported that having a distorted body image was associated with weight control behaviors among preadolescents [16, 21] and adolescents [22–24]. For example, Chung et al. reported that self-perceived overweight/obesity was strongly associated with weight loss behaviors among children and adolescents [21]. Moreover, Liechty et al. reported that non-overweight children and adolescents with a distorted body image (overestimation of weight status) were at a significantly greater risk of initiating unnecessary and unsafe weight loss behavior than those without a distorted body image [24]. Unhealthy weight control behaviors, including skipping meals, eating very little, and using food substitutes and diet pills, have also been reported to be associated with increased weigh gain over time [25, 26]. However, no information regarding weight control behaviors was collected in the present study. Therefore, it will be necessary to consider the effect of weight control behaviors on the association between self-perception of being overweight and the development of overweight in future studies.

In this study, the students who perceived themselves as thin at baseline were at greater risk to being underweight 3 years later than those who perceived themselves as normal weight, among boys and girl, separately. This association persisted even after controlling for lifestyle factors at baseline. Duong et al. reported that perceived weight status among children and adolescents (age 11–17 years) predicted weight status 6 years later, and those who perceived themselves as thin were less likely to be overweight/obese later in USA [12]. Among girls, the preference for an extremely slim body might be rooted in a lack of proper understanding regarding average body weight, which might also be affected by the fact that they set their ideal shape at a level even lower than their misunderstood average [27]. In contrast, by adolescence, boys may be increasingly concerned with becoming more muscular [28]. However, to the best of our knowledge, no other studies have been conducted on the effect of perceived underweight in fourth-grade students on underweight in seventh-grade students among boys. Therefore, further studies will be needed to investigate this association.

This was the first population-based study to prospectively investigate the association between distorted body image and the development of overweight or underweight among normal weight preadolescents for each sex. The strengths of this study include the high participation (over 99 %) and follow-up rates (about 90 %). In addition, self-perceived weight status was not affected by the student’s actual weight status, because the questionnaire survey was conducted before anthropometric measurements. Moreover, most studies that have been conducted on body image among Japanese children and adolescents have used self-reported weight and height to calculate BMI, which has been reported to be inaccurate [29]; however, in this study, we actually measured the height and weight of over 2000 schoolchildren.

Nonetheless, the present study did have a few limitations. First, this study did not consider sociocultural factors [23] and psychological factors [30, 31]. For instance, Xie et al. reported that sociocultural factors, such as parental education, media, and attitudes towards physical appearance was associated with adolescents’ weight perception, in China in 2002 [23]. Moreover, Tang J et al. reported that perceived weight status was associated with psychological symptoms, such as depressive symptoms and anxiety symptoms, in school adolescents in China in 2007 [30]. Thus, sociocultural factors and psychological factors might influence perceived weight status. Second, although we investigated the relationship between distorted body image at baseline and overweight or underweight 3 years later, body image at baseline might be different from that 3 years later. Therefore, further studies will be required to consider the change of body image from baseline to 3 years later. Third, the results of the present study might be affected by a health promotion program for the prevention of childhood lifestyle related diseases in the study area. Finally, the participants in this prospective cohort study were fourth-grade students (age 9–10 years) from only one town in Japan; this might limit the generalizability of the results to other Japanese schoolchildren or populations. Therefore, further longitudinal studies will be required to confirm the present study results in preadolescents from other areas.

## Conclusions

The results of the present study suggest that self-perceived weight status among normal weight preadolescents is associated with being overweight or underweight in adolescence. Students who perceived themselves as heavy were at a greater risk of being overweight than those who perceived themselves as normal, among boys and girls, separately. In addition, those who perceived themselves as thin were at a greater risk of being underweight than those who perceived themselves as normal, among boys and girls, separately. Therefore, improved education on self-perceived weight status among preadolescents in Japan could help prevent the development of underweight and overweight/obesity.

## Abbreviations

95 % CI, 95 % confidence interval; BMI, body mass index; ORs, odds ratios; SD, standard deviation
